# The impact of pediatric constipation on appendicitis: a prospective birth cohort in the Japan Environment and Children’s Study

**DOI:** 10.1186/s12887-025-06263-7

**Published:** 2025-11-26

**Authors:** Hiroyoshi Iwata, Takeshi Yamaguchi, Sachiko Itoh, Yu Ait Bamai, Atsuko Ikeda, Keitaro Makino, Mariko Itoh, Maki Tojo, Rieko Yamamoto, Naomi Tamura, Rahel Mesfin Ketema, Yasuaki Saijo, Yoshiya Ito, Reiko Kishi, the Japan Environment and Children’s Study (JECS) Group, Michihiro Kamijima, Shin Yamazaki, Maki Fukami, Chiharu Ota, Koichi Hashimoto, Chisato Mori, Shuichi Ito, Ryoji Shinohara, Hidekuni Inadera, Takeo Nakayama, Ryo Kawasaki, Yasuhiro Takeshima, Seiji Kageyama, Narufumi Suganuma, Shoichi Ohga, Takahiko Katoh

**Affiliations:** 1 Center for Environmental and Health Sciences, Hokkaido University, North-12, West-7, Kita-Ku, Sapporo, 060-0812 Japan; 2 Faculty of Health Sciences, Hokkaido University, North-12, West-5, Kita-Ku, Sapporo, 060-0812 Japan; 3 Department of Social Medicine, Asahikawa Medical University, 1-1-1 Midorigaoka-Higashi-2-Jo, Asahikawa, 078-8510 Japan; 4 Faculty of Nursing, Japanese Red Cross Hokkaido College of Nursing, 664-1 Akebono-Cho, Kitami, 090-0011 Japan

**Keywords:** Constipation, Appendicitis, Japan Environment and Children’s Study

## Abstract

**Background:**

This study aimed to examine the association between constipation and appendicitis in children using data from a nationwide birth cohort, the Japan Environment and Children's Study (JECS).

**Methods:**

Data were obtained from 64,772 children and their mothers participating in JECS, which included over 100,000 pregnancies across 15 regional centres in Japan. Pediatric constipation was assessed at age three using the Rome III criteria, while appendicitis cases were identified between ages three to four. Logistic regression analyses, including univariable and multivariable models, were conducted to evaluate associations. Additional analyses were stratified by sex.

**Results:**

Among the 64,772 children analyzed, 156 cases of appendicitis were identified between the ages of 3 and 4 years. Logistic regression suggested a possible association between constipation and an increased risk of appendicitis (univariable odds ratio [OR]: 1.38, 95% confidence interval [CI]: 0.87–2.10; multivariable OR: 1.36, 95% CI: 0.86–2.06). Although the associations did not reach statistical significance, the point estimates consistently indicated a trend toward increased risk.

**Conclusion:**

Although our analyses of children aged 3–4 years did not yield statistically significant associations, the observed trends may suggest a potential link between constipation and appendicitis. These findings should be interpreted only as exploratory, and further studies in different age groups, including older children and adults, are warranted to confirm or refute this possible association.

**Supplementary Information:**

The online version contains supplementary material available at 10.1186/s12887-025-06263-7.

## Background

Appendicitis is a global health concern, often requiring surgical intervention. In 2021, an estimated 2,193,020 new cases of appendicitis occurred in children, representing 12.9% of all appendicitis cases in the general population worldwide [1]. The overall lifetime risk of acute appendicitis is reported to be from 7–8% [2, 3]. In 2021, pediatric appendicitis accounted for approximately 10% of all cases of appendicitis in the general population [1]. The risk of developing appendicitis is generally higher during adolescence than during preschool [4]. However, pediatric appendicitis requires careful attention. Children under 6 years old often present with advanced appendicitis due to nonspecific symptoms and delayed diagnosis, with more than half of cases classified as complicated [4, 5]. Therefore, identifying risk factors for pediatric appendicitis and emphasizing prevention are crucial for public health and child well-being.

The mechanisms underlying appendicitis remain unclear. Etiologically, obstruction of the appendiceal lumen by any mechanism is considered to promote bacterial overgrowth, leading to acute inflammation and abscesses [6]. Moreover, the underlying causes of luminal obstruction may differ by age group, underscoring the need for age-specific consideration of appendicitis [6]. Consequently, constipation, which can obstruct the appendiceal lumen, should be recognized as a potential risk factor. Constipation is common in children, with the prevalence of functional constipation estimated to be approximately 9.5–25% worldwide [7, 8]. However, in Japan, Fujitani et al. reported that 20% of children aged 3–8 years had functional constipation in Yokohama city, Japan [9].

Although several risk factors for pediatric appendicitis have been proposed, such as sex and socioeconomic status (SES), constipation has not been well investigated [10]. To our knowledge, evidence supporting constipation as a risk factor for appendicitis in children is limited. Mahajan et al., analyzing a large database, showed that pediatric patients with constipation and abdominal pain were more likely to have missed appendicitis, with an adjusted odds ratio (OR) of 2.43 and 95% confidence interval (CI) of 1.86–3.17 [11]. Conversely, dietary fiber intake, a remedy for constipation, has been reported to be associated with a low incidence of appendicitis [12, 13]. These findings support the positive association between constipation and appendicitis.

However, previous analyses have not demonstrated an association between constipation and appendicitis in an epidemiological cohort study. Epidemiological studies are considered the most suitable approach for collecting information on pre-disease occurrence in the general population and assessing risk by comparing cases with non-cases among a large number of research participants. We focused on children aged 3–4 years because appendicitis at this age carries a higher risk of perforation, symptoms are often difficult for children to communicate, and constipation can be reliably assessed using validated caregiver questionnaires. Based on these considerations, this study aimed to investigate the association between constipation at 3–4 years of age and the subsequent incidence of pediatric appendicitis using data from a large nationwide prospective birth cohort in Japan.

## Methods

### Study design and population

**Fig. 1:**
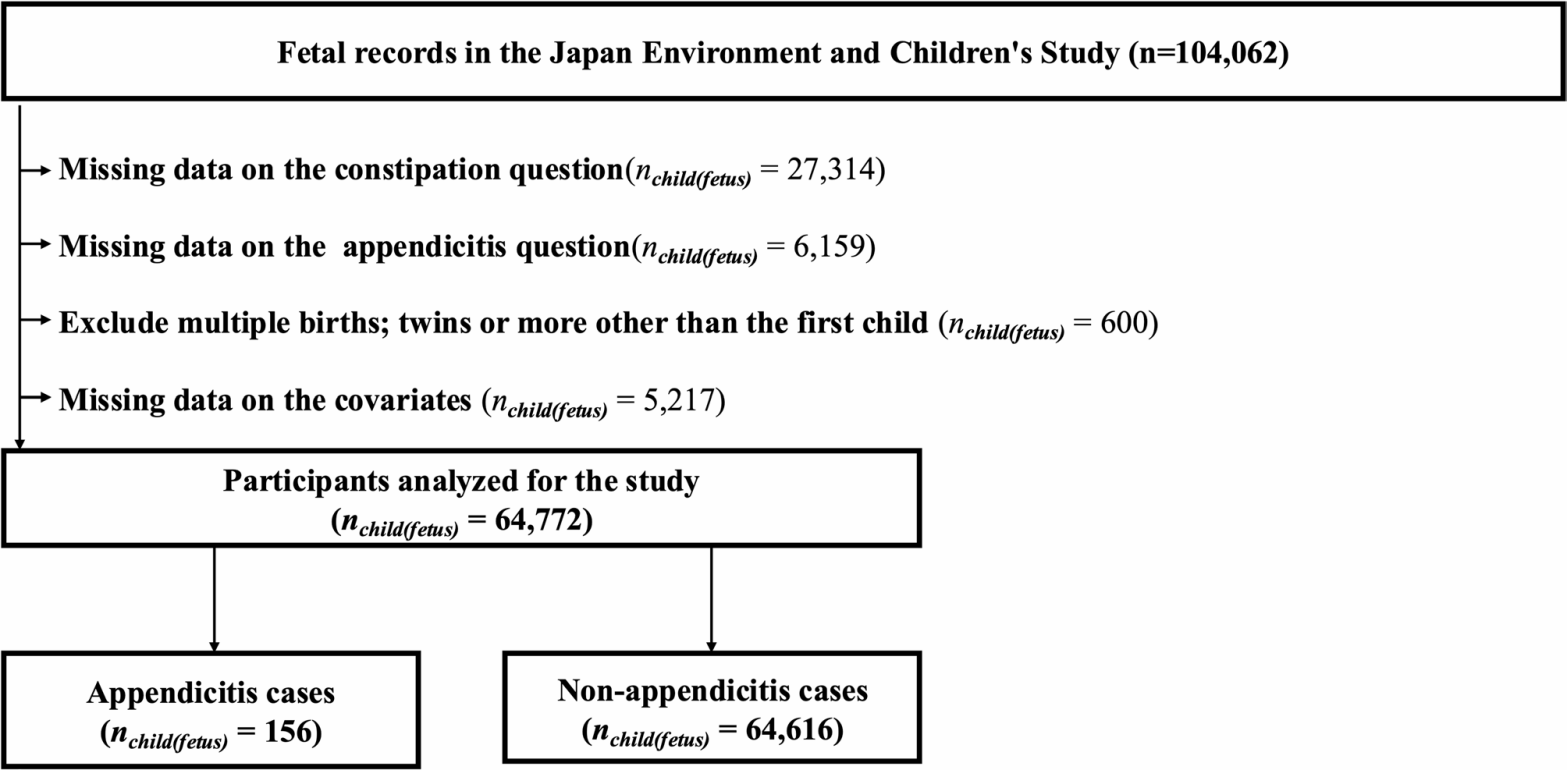
Study participant inclusion flow chart

This analysis was conducted within the Japan Environment and Children’s Study (JECS), an ongoing nationwide prospective birth cohort in Japan. It represents a sub-analysis of the JECS, focusing on data collected when the participating children were 3–4 years of age to examine the cross-sectional association between constipation and the subsequent incidence of pediatric appendicitis. The JECS recruited pregnant mothers from 15 Regional Centres across Japan between 2011 and 2014, including more than 100,000 pregnancies (jecs-ta-20190930 and jecs-qa-20210401 datasets). The detailed JECS methodology has been described by Kawamoto et al. [14]. The JECS covers approximately half of all live births in the Study Area [15, 16]. For the present analysis, we included children whose caregivers completed the 3–4 years of age questionnaire. We excluded participants with missing responses to items on constipation or appendicitis, those with missing data on key covariates, and those from twin or higher-order multiple births. A flowchart of participant inclusion and exclusion is shown in Fig. 1. In accordance with the STROBE criteria for observational studies, we have ensured that the study design and reporting adhere to these guidelines. The completed STROBE checklist is provided as Additional file 1 [17, 18].

### Assessment of pediatric constipation

The present study collected data from caregivers using the pediatric constipation Rome III when the children were 3 years old [19–23]. “Two or fewer defecations per week (Question 1),” “At least 1 episode per week of fecal incontinence after the acquisition of toileting skills (Question 2),” “History of excessive stool retention (Question 3),” “History of painful or hard bowel movements (Question 4),” “Presence of a large fecal mass in the rectum (Question 5),” “History of large-diameter stools that may obstruct the toilet (Question 6)” [21]. These six questions were evaluated. Furthermore, we assessed “composite constipation,” which met at least two of the six above questions [19–22]. In the JECS, caregiver questionnaires were administered prospectively every 6 months to 1 year, which minimized the potential for recall bias and ensured that constipation was assessed in a timely manner during early childhood.

### Assessment of pediatric appendicitis

The primary outcome was the incidence of pediatric appendicitis during the past one-year period from age three to four. Caregivers were asked to complete parent-reported questionnaires distributed by the JECS once or twice annually. At the 4-year survey, the questionnaire included an item on the incidence of physician-diagnosed pediatric appendicitis within the preceding year.

### Statistical analysis

First, descriptive statistics were used to evaluate the basic characteristics of the mothers and their children. Next, univariable and multivariable logistic regression analyses were performed, and the outcome was the incidence of pediatric appendicitis. Six questions on constipation and the presence of composite constipation were used as exposure-independent variables. Variables with missing values were excluded before conducting the regression analyses. We defined *P*-values < 0.05 as statistically significant. Statistical analyses were performed using the R software (version 4.0.3; R Foundation for Statistical Computing, Vienna, Austria).

### Covariates

**Fig. 2:**
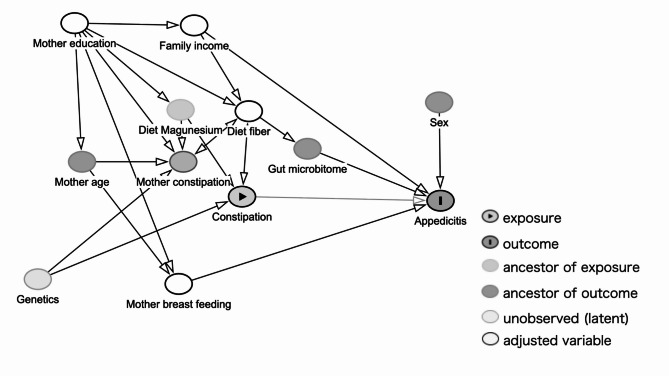
Directed acyclic graph (DAG) depicting the conceptual framework of the present study. i. Exposure variable: child constipation. ii. Outcome variable: child appendicitis. iii. Adjusted variable: maternal breastfeeding, family income, and diet fiber amount

Covariates were selected using a directed acyclic graph (DAG) based on clinical expertise, knowledge, and importance. The DAG was constructed with "Dagitty” [24] (Fig. 2). Given the confounder effect in our DAG, we identified the following potential covariates: child sex, diet fiber intake, mother breastfeeding, and family income. Recently, SES has been considered a risk factor for pediatric appendicitis; thus, family income was used as a proxy for SES in our analysis [10].

### Covariate definitions

The JECS used questionnaires to ask mothers about breastfeeding at one month postpartum [25]. Maternal dietary intake during pregnancy was assessed using food frequency questionnaires (FFQs), and total daily fiber intake was calculated [26, 27]. Family income was categorized into two strata for analysis: low income (annual income < 4 million yen) and moderate or higher income (annual income ≥ 4 million yen), given the average income per household is in the region of 4 million yen at the median [28]. As JECS does not collect postnatal dietary data, maternal dietary data during pregnancy were used as a proxy for the children’s diet.

### Additional analysis

Since previous studies have reported that male children tend to suffer from appendicitis more than female children [10], the whole group was stratified by sex, and univariable and multivariable regressions were conducted within each group.

### Sample size calculation

Sample size was estimated based on the event-per-variable rule of 10 [29]. Given that our multivariable regression model included three or more variables, at least 30 appendicitis events were required.

In addition, a two-sample proportions approach was applied using Stata (version 17.0; StataCorp LLC, College Station, TX, USA), following the method recommended by Serdar et al. [30]. Assuming a baseline annual incidence of pediatric appendicitis in preschool children of 0.00036 (0.036%) [31] and a constipation prevalence of 25% [7], the required total sample sizes to achieve 80% power at a two-sided 5% significance level were approximately 63,216 for OR = 3.0, 97,360 for OR = 2.5, 185,108 for OR = 2.0, 335,980 for OR = 1.7, 603,676 for OR = 1.5, and 1,523,816 for OR = 1.3. When applying a constipation prevalence of 20% [9], the required sample sizes further increased, reaching approximately 75,030 for OR = 3.0, 115,440 for OR = 2.5, 219,105 for OR = 2.0, 397,025 for OR = 1.7, 712,235 for OR = 1.5, and 1,794,100 for OR = 1.3.

## Results

Descriptive analyses are summarized in Table 1. Among the 64,772 children, the primary outcome, pediatric appendicitis during the 1-year period from age 3 to 4, occurred in 156 cases (0.24%).　Male children were more frequent overall, and the proportion of males was higher in the appendicitis group than in the non-appendicitis group. However, a higher proportion of males was observed in the appendicitis group. Higher proportions of moderate or high family income, mother’s education duration over 12 years, and mother’s breastfeeding were observed in the non-appendicitis group. The distribution of the Rome III individual questions and the integrated constipation assessment are summarized in Table 1. The integrated constipation assessment had a higher proportion in the appendicitis group than in the non-appendicitis group.

**Table 1:** Study participant characteristics

	Appendicitis group	Non-appendicitis group	Overall	*P*-value
	(*N* = 156)	(*N* = 64,616)	(*N* = 64,772)	
Child sex				
Female	71 (45.5%)	31,778 (49.2%)	31,849 (49.2%)	
Male	85 (54.5%)	32,838 (50.8%)	32,923 (50.8%)	0.40*
Mother fiber consumption at pregnancy, g/day				
Median [Q1, Q3]	9.55 [7.30, 13.2]	10.3 [7.60, 13.8]	10.3 [7.60, 13.8]	0.18**
Family income				
Moderate or higher income family	80 (51.3%)	39,910 (61.8%)	39,990 (61.7%)	
Low income family	76 (48.7%)	24,706 (38.2%)	24,782 (38.3%)	0.01*
Mother education duration over 12 years or less				
Positive	73 (46.8%)	42,946 (66.5%)	43,019 (66.4%)	
Negative	83 (53.2%)	21,670 (33.5%)	21,753 (33.6%)	< 0.001*
Mother breastfeeding within 1 month of birth				
Positive	80 (51.3%)	35,280 (54.6%)	35,360 (54.6%)	
Negative	76 (48.7%)	29,336 (45.4%)	29,412 (45.4%)	0.45*
Question 1: Two or fewer defecations per week				
Positive	6 (3.8%)	2755 (4.3%)	2761 (4.3%)	
Negative	150 (96.2%)	61,861 (95.7%)	62,011 (95.7%)	0.95*
Question 2: At least 1 episode per week of fecal incontinence after the acquisition of toileting skills				
Positive	15 (9.6%)	4371 (6.8%)	4386 (6.8%)	
Negative	141 (90.4%)	60,245 (93.2%)	60,386 (93.2%)	0.21*
Question 3: History of excessive stool retention				
Positive	12 (7.7%)	5089 (7.9%)	5101 (7.9%)	
Negative	144 (92.3%)	59,527 (92.1%)	59,671 (92.1%)	1.00*
Question 4: History of painful or hard bowel movements				
Positive	39 (25.0%)	13,002 (20.1%)	13,041 (20.1%)	
Negative	117 (75.0%)	51,614 (79.9%)	51,731 (79.9%)	0.16*
Question 5: Presence of a large fecal mass in the rectum				
Positive	10 (6.4%)	4049 (6.3%)	4059 (6.3%)	
Negative	146 (93.6%)	60,567 (93.7%)	60,713 (93.7%)	1.00*
Question 6: History of large-diameter stools that may obstruct the toilet				
Positive	4 (2.6%)	1726 (2.7%)	1730 (2.7%)	
Negative	152 (97.4%)	62,890 (97.3%)	63,042 (97.3%)	1.00*
Integrated constipation assessment				
Positive	24 (15.4%)	7504 (11.6%)	7528 (11.6%)	
Negative	132 (84.6%)	57,112 (88.4%)	57,244 (88.4%)	0.18*

^*^Chi-square test

^**^Wilcoxon rank-sum test

Univariable and multivariable regression analyses for pediatric appendicitis are presented in Table 2. In the multivariable analyses, all models were adjusted for maternal breastfeeding, family income, and dietary fiber intake, as specified in the DAG. The univariable regression for “Question 2: At least 1 episode per week of fecal incontinence after the acquisition of toileting skills” showed an odds ratio of 1.47 and 95% CI [0.83–2.41]. The univariable regression for “Question 4: History of painful or hard bowel movements ' showed an odds ratio of 1.32 and 95% CI [0.91–1.88]. The integrated constipation assessment had an odds ratio of 1.38 and a 95% CI [0.87–2.10]. Other questions showed odds ratios above 1.0, except for “Question 3: History of excessive stool retention” and “Question 6: History of large-diameter stools that may obstruct the toilet”. Multivariable regression analyses, adjusted for child sex, dietary fiber intake, mother breastfeeding, and family income, showed an odds ratio of 1.46 with 95% CI [0.82–2.41] and 1.30 with 95% CI [0.89–1.85] for “Questions 2 and 4,” respectively. The integrated constipation assessment in the multivariable analysis had an odds ratio of 1.36 with 95% CI [0.86–2.06].

**Table 2:** Univariable and multivariable regression results

Univariable analysis	Odds ratio	Lower 95%CI*	Upper 95%CI*	*P*-value	Multivariable analysis*	Odds ratio	Lower 95%CI*	Upper 95%CI*	*P*-value
Question 1: Two or fewer defecations per week	0.9	0.35	1.86	0.80	Question 1: Two or fewer defecations per week	0.88	0.35	1.82	0.76
Question 2: At least 1 episode per week of fecal incontinence after the acquisition of toileting skills	1.47	0.83	2.41	0.16	Question 2: At least 1 episode per week of fecal incontinence after the acquisition of toileting skills	1.46	0.82	2.41	0.16
Question 3: History of excessive stool retention	0.98	0.51	1.68	0.93	Question 3: History of excessive stool retention	0.96	0.51	1.66	0.90
Question 4: History of painful or hard bowel movements	1.32	0.91	1.88	0.13	Question 4: History of painful or hard bowel movements	1.3	0.89	1.85	0.16
Question 5: Presence of a large fecal mass in the rectum	1.03	0.5	1.85	0.94	Question 5: Presence of a large fecal mass in the rectum	1.01	0.5	1.82	0.98
Question 6: History of large-diameter stools that may obstruct the toilet	0.96	0.3	2.27	0.93	Question 6: History of large-diameter stools that may obstruct the toilet	0.95	0.29	2.25	0.92
Integrated constipation assessment	1.38	0.87	2.1	0.14	Integrated constipation assessment	1.36	0.86	2.06	0.17

*Abbreviations:* CI Confidence interval

^*^All multivariable models were adjusted for maternal breastfeeding, family income, and dietary fiber intake, as pre-specified in the DAG

Additional regression analyses were conducted and stratified by the child’s sex (Additional file 2). In female children, the multivariable regression results show “Question 2” and ''Question 4” with odds ratios of 1.60 with 95% CI [0.67–3.26], and 1.30 with 95% CI [0.76–2.15], respectively. In male children, the multivariable regression results show “Question 2” and “Question 4'' with odds ratios of 1.35 with 95% CI [0.60–2.62] and 1.32 with 95% CI [0.77–2.16], respectively.

## Discussion

We analyzed data from the JECS, which originally enrolled 104,062 pregnancies, and included 64,772 children with complete data in the present analysis. Among children aged 3–4 years, there were no statistically significant associations between constipation and pediatric appendicitis; therefore, the findings should be interpreted only as exploratory trends rather than definitive evidence. Nonetheless, our regression analyses indicated that the integrated constipation assessment from the Rome III criteria was positively associated with pediatric appendicitis. In addition, sex-stratified analyses revealed that the questions with statistical significance varied. In the sex-stratified analyses, although none of the Rome III items reached statistical significance, certain items (e.g., items 1, 3, and 5) showed tendencies toward differential associations between boys and girls. These patterns should not be overinterpreted but regarded as suggestive signals requiring validation.

There are several possible explanations for the absence of a clear statistical association in this study. First, pediatric-specific challenges in symptoms and diagnosis may have played a role. Constipation in young children cannot always be accurately detected by stool frequency alone, and the clinical presentation of appendicitis in preschool children is often atypical and less specific. Previous studies and reviews have consistently highlighted that appendicitis in children under 5 years remains difficult to diagnose despite the availability of advanced imaging, partly due to unusual symptoms and the limited ability of younger children to describe pain [10, 32, 33]. These diagnostic limitations may have influenced our findings and contributed to the uncertainty observed in this age group.

Furthermore, our recalculated sample size estimates indicated that although we had secured the minimum number of participants required, the study was still likely underpowered to detect a statistically significant association. Given the very low baseline incidence of pediatric appendicitis and the relatively low prevalence of constipation (9.5–25% globally and 20% in Japan), extremely large cohorts would be required to attain adequate statistical power. This limitation suggests that the lack of statistical significance in our findings may have been due more to sample size constraints than to the true absence of an association. Although future research should consider even larger cohorts, it is important to recognize that the JECS already represents one of the world’s largest birth cohorts. Therefore, collaborative studies that leverage domestic and international cohort networks will be essential to more definitively clarify this relationship.

There are three key developmental periods associated with the onset of constipation during childhood, including 1) the transition from breast milk to formula and the introduction of solid foods in infants, 2) toilet training during toddler years, and 3) the start of school and avoidance of defecation at school in school-aged children [34–36]. Among them, the peak incidence of constipation is believed to occur during the toilet training period between the ages 2 and 4 [34, 36]. Thus, our analyses covered only the early stage of child constipation. Further studies are needed to evaluate other age groups and adults.

Our study did not demonstrate a statistically significant association between childhood constipation and appendicitis. Nevertheless, constipation can mimic appendicitis symptoms, which remains a diagnostic challenge for clinicians [37]. Beyond appendicitis, childhood constipation itself is an important public health issue, as it adversely affects quality of life and contributes to healthcare utilization [8, 38, 39].

Given the observed positive association between childhood constipation and appendicitis, some clinical and public health suggestions can be considered. From a clinical perspective, physicians care for a tremendous number of children with abdominal pain or fever who are suspected of having appendicitis. Incorporating the Rome III criteria, questions 2 or 4, or the existence of constipation, would aid in the diagnosis of appendicitis. However, clinicians often face challenges in differentiating between acute constipation and acute appendicitis due to their symptomatic similarities [37]. From a public health perspective, our results emphasize the importance of preventing childhood constipation. The complications of child constipation extend beyond the risk of appendicitis and can also lead to various symptoms that are not related to the gastrointestinal system and significantly impact the quality of life for children, often underappreciated by healthcare systems [8, 39]. In addition, a large amount of public funds has been spent on caring for children with constipation due to regular clinic visits, repeated emergency room visits, and hospital admissions [8, 38].

Our study has some limitations. First, although the Rome IV criteria have replaced the Rome III criteria as the latest standards for diagnosing functional constipation [40], the timing of data collection coincided with the Rome III era. Second, the questionnaires were similar. The assessment was based on questionnaires rather than evaluations from medical records. Third, total dietary fiber intake was estimated using maternal FFQ rather than direct dietary assessments of the children [27], limiting precision. Fourth, our analyses could not adjust for unmeasured confounders, such as the gut microbiome, laxative use, or other medication exposure. Fifth, the appendicitis diagnosis was based on parental reports of physician diagnosis without verification from surgical or histological records, which may have led to potential misclassification. Moreover, treatment information was not available in the present study, making it difficult to assess whether early management of constipation could reduce constipation-related complications and potentially lower the risk of appendicitis, an important area for future research. Sixth, previous studies have reported racial and ethnic variations in the incidence and clinical presentation of appendicitis, which may reflect differences in genetics, healthcare access, and health-seeking behaviors [41–43]. As our cohort consists solely of Japanese children, the generalizability of our findings to more diverse populations may be limited.

Appendicitis cases in our study were based on caregiver-reported physician diagnoses from annual questionnaires. We could not confirm histopathological diagnoses or differentiate true appendicitis from possible negative appendectomies, which may have led to some degree of misclassification. Another limitation is the relatively small number of appendicitis cases, which may have limited the statistical power and resulted in unstable effect estimates. Additionally, constipation was assessed using the Rome III questionnaire, whereas the currently used Rome IV criteria require symptoms to persist for at least 1 month instead of 2 months as in Rome III [40]. Therefore, children with relatively shorter but clinically relevant episodes of constipation may have been missed in our study, potentially leading to an underestimation of constipation prevalence and attenuation of the observed associations. Nevertheless, a study by Russo et al. (mean age 77.4 months, approximately 6.5 years) reported no substantial difference between Rome III and Rome IV criteria in estimating constipation prevalence [44], suggesting that the impact of this difference on our findings may be limited.

## Conclusions

We assessed the potential association between pediatric constipation and appendicitis in children aged 3–4 years using a large prospective birth cohort in Japan. Although some Rome III criteria questions and integrated constipation assessment analyses suggested possible positive associations with appendicitis, these findings did not reach statistical significance and should, therefore, be regarded only as exploratory. Our results may provide preliminary insights that could inform future research on early pediatric appendicitis diagnosis. We hope that further studies, including those in children of other age groups and adults, will clarify this association.

## Supplementary Information

Additional file 1. STROBE checklist

Additional file 2. Univariable and multivariable regression results stratified by child sex

## Data Availability

Data are unsuitable for public deposition due to ethical restrictions and legal framework of Japan. It is prohibited by the Act on the Protection of Personal Information (Act No. 57 of 30 May 2003, amendment on 9 September 2015) to publicly deposit the data containing personal information. Ethical Guidelines for Medical and Health Research Involving Human Subjects enforced by the Japan Ministry of Education, Culture, Sports, Science and Technology and the Ministry of Health, Labour and Welfare also restricts the open sharing of the epidemiologic data. All inquiries about access to data should be sent to: jecs-en@nies.go.jp. The person responsible for handling enquiries sent to this e-mail address is Dr Shoji F. Nakayama, JECS Programme Office, National Institute for Environmental Studies.

## Abbreviations

JECS: Japan Environment and Children's Study
FFQ: Food frequency questionnaire
DAG: Directed acyclic graph
SES: Socioeconomic status
