# Supplementary material for: The impact of pediatric constipation on appendicitis: a prospective birth cohort in the Japan Environment and Children’s Study
Source: BMC Pediatr. 2025 Nov 26;25:962. doi: 10.1186/s12887-025-06263-7 (PMC12649063; doi:10.1186/s12887-025-06263-7)
Supplement: Supplementary file 2 — Additional file 2. Univariable and multivariable regression results stratified by child sex [file 12887_2025_6263_MOESM2_ESM.docx]

**Table S2**  Univariable and multivariable regression results stratified by child sex.

| Female |  |  |  |  |  |  |  |  |  |
| --- | --- | --- | --- | --- | --- | --- | --- | --- | --- |
| Univariable analysis | Odds Ratio | Lower 95% CI | Upper 95% CI | P-value | Multivariable analysis** | Odds Ratio | Lower 95% CI | Upper 95% CI | P-value |
| Question 1: Two or fewer defecations per week | 1.53 | 0.54 | 3.44 | 0.36 | Question 1: Two or fewer defecations per week | 1.47 | 0.51 | 3.31 | 0.41 |
| Question 2: At least 1 episode per week of fecal incontinence after the acquisition of toileting skills | 1.61 | 0.67 | 3.28 | 0.23 | Question 2: At least 1 episode per week of fecal incontinence after the acquisition of toileting skills | 1.60 | 0.67 | 3.26 | 0.24 |
| Question 3: History of excessive stool retention | 1.20 | 0.50 | 2.45 | 0.64 | Question 3: History of excessive stool retention | 1.18 | 0.49 | 2.40 | 0.68 |
| Question 4: History of painful or hard bowel movements | 1.34 | 0.78 | 2.22 | 0.26 | Question 4: History of painful or hard bowel movements | 1.30 | 0.76 | 2.15 | 0.32 |
| Question 5: Presence of a large fecal mass in the rectum | 1.46 | 0.61 | 2.97 | 0.34 | Question 5: Presence of a large fecal mass in the rectum | 1.42 | 0.59 | 2.90 | 0.38 |
| Question 6: History of large-diameter stools that may obstruct the toilet | 1.01 | 0.17 | 3.22 | 0.99 | Question 6: History of large-diameter stools that may obstruct the toilet | 0.99 | 0.16 | 3.17 | 0.99 |
| Integrated constipation assessment | 1.69 | 0.90 | 2.94 | 0.08 | Integrated constipation assessment | 1.63 | 0.87 | 2.85 | 0.10 |
|  |  |  |  |  |  |  |  |  |  |
| Male |  |  |  |  |  |  |  |  |  |
| "Univariable analysis " | Odds Ratio | Lower 95% CI | Upper 95% CI | P-value | Multivariable analysis** | Odds Ratio | Lower 95% CI | Upper 95% CI | P-value |
| Question 1: Two or fewer defecations per week | 0.30 | 0.02 | 1.35 | 0.23 | Question 1: Two or fewer defecations per week | 0.30 | 0.02 | 1.34 | 0.23 |
| Question 2: At least 1 episode per week of fecal incontinence after the acquisition of toileting skills | 1.35 | 0.60 | 2.62 | 0.42 | Question 2: At least 1 episode per week of fecal incontinence after the acquisition of toileting skills | 1.35 | 0.60 | 2.62 | 0.42 |
| Question 3: History of excessive stool retention | 0.78 | 0.27 | 1.74 | 0.59 | Question 3: History of excessive stool retention | 0.78 | 0.27 | 1.73 | 0.58 |
| Question 4: History of painful or hard bowel movements | 1.34 | 0.78 | 2.18 | 0.27 | Question 4: History of painful or hard bowel movements | 1.32 | 0.77 | 2.16 | 0.29 |
| Question 5: Presence of a large fecal mass in the rectum | 0.62 | 0.15 | 1.65 | 0.41 | Question 5: Presence of a large fecal mass in the rectum | 0.61 | 0.15 | 1.64 | 0.40 |
| Question 6: History of large-diameter stools that may obstruct the toilet | 0.92 | 0.15 | 2.92 | 0.91 | Question 6: History of large-diameter stools that may obstruct the toilet | 0.92 | 0.15 | 2.90 | 0.90 |
| Integrated constipation assessment | 1.13 | 0.55 | 2.09 | 0.72 | Integrated constipation assessment | 1.12 | 0.54 | 2.07 | 0.73 |

*CI; Confidence interval.

**All multivariable models were adjusted for maternal breastfeeding, family income, and dietary fiber intake, as pre-specified in the DAG.
